# Metagenome-Assembled Genomes From *Pyropia haitanensis* Microbiome Provide Insights Into the Potential Metabolic Functions to the Seaweed

**DOI:** 10.3389/fmicb.2022.857901

**Published:** 2022-03-23

**Authors:** Junhao Wang, Xianghai Tang, Zhaolan Mo, Yunxiang Mao

**Affiliations:** ^1^Key Laboratory of Marine Genetics and Breeding (Ministry of Education), College of Marine Life Sciences, Ocean University of China, Qingdao, China; ^2^Key Laboratory of Tropical Aquatic Germplasm of Hainan Province, Sanya Oceanographic Institution, Ocean University of China, Sanya, China; ^3^Key Laboratory of Utilization and Conservation of Tropical Marine Bioresource (Ministry of Education), College of Fisheries and Life Sciences, Hainan Tropical Ocean University, Sanya, China; ^4^Yazhou Bay Innovation Research Institute, Hainan Tropical Ocean University, Sanya, China; ^5^Key Laboratory for Conservation and Utilization of Tropical Marine Fishery Resources of Hainan Province, Hainan Tropical Ocean University, Sanya, China

**Keywords:** *Pyropia haitanensis*, microbial community, metagenome, binning, microbial metabolic function

## Abstract

*Pyropia* is an economically important edible red alga worldwide. The aquaculture industry and *Pyropia* production have grown considerably in recent decades. Microbial communities inhabit the algal surface and produce a variety of compounds that can influence host adaptation. Previous studies on the *Pyropia* microbiome were focused on the microbial components or the function of specific microbial lineages, which frequently exclude metabolic information and contained only a small fraction of the overall community. Here, we performed a genome-centric analysis to study the metabolic potential of the *Pyropia haitanensis* phycosphere bacteria. We reconstructed 202 unique metagenome-assembled genomes (MAGs) comprising all major taxa present within the *P. haitanensis* microbiome. The addition of MAGs to the genome tree containing all publicly available *Pyropia*-associated microorganisms increased the phylogenetic diversity by 50% within the bacteria. Metabolic reconstruction of the MAGs showed functional redundancy across taxa for pathways including nitrate reduction, taurine metabolism, organophosphorus, and 1-aminocyclopropane-1-carboxylate degradation, auxin, and vitamin B_12_ synthesis. Some microbial functions, such as auxin and vitamin B_12_ synthesis, that were previously assigned to a few *Pyropia*-associated microorganisms were distributed across the diverse epiphytic taxa. Other metabolic pathways, such as ammonia oxidation, denitrification, and sulfide oxidation, were confined to specific keystone taxa.

## Introduction

Marine algae are an ancient and functionally important component of aquatic ecosystems (Raven et al., 2002; Armbrust et al., 2004; Brodie et al., 2017b), providing valuable sources of food and habitat for a variety of marine microorganisms and animals (Seymour et al., 2017). Similar to the rhizosphere, microbes inhabit the “phycosphere” (Cole, 1982) surrounding seaweed-based cells, which extend from the cell surface through the dispersal boundary layer of seaweed-derived dissolved organic compounds, secondary metabolites, and exopolymeric substances (Bell and Mitchell, 1972). Interactions between algae and microbes are thought to occur in this specific environment (Brodie et al., 2017a). In recent years, many phycologists and microbiologists have explored potential interactions between algae and microbes (Wichard, 2015; Aranda et al., 2016; Antunes et al., 2019). The currently accepted hypothesis is that microorganisms have beneficial, neutral, and detrimental effects on algae, with beneficial effects being the most widely studied (Amin et al., 2015; van Tol et al., 2017). Algae-associated bacteria are involved in the exchange of diverse chemical currencies, including vitamins (Croft et al., 2005; Xie et al., 2013), hormones (Amin et al., 2015), quorum sensing signals (Geng and Belas, 2010; Huang et al., 2018), dissolved organic carbon (Teeling et al., 2012), and nutrient remineralization (Klawonn et al., 2019). These studies have mainly been conducted on some ecologically important phytoplankton (Geng and Belas, 2010; Xie et al., 2013; Buchan et al., 2014; Amin et al., 2015; Huang et al., 2018; Foster and Zehr, 2019; Nowinski et al., 2019; Rambo et al., 2020) and macroalgae species (Twigg et al., 2014; Vollmers et al., 2017; Roth-Schulze et al., 2018), and only few studies on farmed seaweed, such as *Pyropia* (Wang et al., 2021).

*Pyropia* is an edible red alga (phylum: Rhodophyta; class: Bangiophyceae), which is mainly consumed as processed food products (Cho and Rhee, 2020). Because *Pyropia* contains high levels of vitamins (e.g., vitamin B_12_), minerals (e.g., iron), and protein contents (Blouin et al., 2011), it is usually regarded as a health-promoting food. In East Asian countries such as China, Japan, and South Korea, *Pyropia* is widely cultivated (Cho and Rhee, 2020) and its production was over 28.7 million tons in 2018 according to FAO statistics (Food and Agriculture Organization [FAO], 2020). Under economic incentives, intensive cultivation breeding and practices increase cultivar susceptibility to diseases and induce colonization and infection of harmful microbes (Yan et al., 2019), leading to the reduced production of *Pyropia*. In contrast, some microorganisms promote algal growth (Amin et al., 2015) and morphological development. Previous studies have shown that marine macroalgae, including *Ulva* (Provasoli and Pintner, 1980; Nakanishi et al., 1996; Marshall et al., 2006), *Monostroma* (Matsuo et al., 2003), and *Pyropia* (Fukui et al., 2014), cannot develop normal morphology under axenic conditions.

Recent efforts continue to explore potential *Pyropia*-microbial interactions in different environments and statuses. For instance, analysis of the epiphytic microorganisms of *P. haitanensis* using amplicon sequencing revealed that the microbial communities differed significantly during various life stages of *P. haitanensis* and hypothesized that microbial taxa could produce some plant growth regulators to promote the growth of *P. haitanensis* (Wang et al., 2020). Moreover, a study on the microbial community of *P. yezoensis* infected with red rot disease revealed the interactions between the disease and the epiphytic and planktonic microbial communities, and the potential of using community differentiation to forecast disease occurrence (Yan et al., 2019). The above studies have made important contributions to the community structure and dynamics of microorganisms in *Pyropia* and have also shown a complex relationship between the host and microbiome. However, these features can only be assigned at taxon level and do not provide information on the functional characteristics of a community. Our previous study on the thalli of laboratory-cultured *P. haitanensis* (PH40) revealed microbial gene functions (Wang et al., 2021). Gene-centric analysis of the whole community has provided critical functional insights into the *Pyropia* microbiome, but could not link function with phylogeny. In this study, we performed a genome-centric analysis to characterize 202 MAGs from the phycosphere of *P. haitanensis* cultivars in China. Our sequenced data represented more than 95% of the entire microbiome. These MAGs could comprise approximately 65% of the sequenced microbes, providing an unprecedented opportunity to determine the metabolic potential of all major taxa within a laver microbiome.

## Materials and Methods

### Sample Collection and DNA Extraction

*P. haitanensis* samples were collected from farming regions in southern and northern China in November 2019. Two farms near coastal water were located in Rizhao City (35°42′N, 119°50′E), Shandong Province, China; and Ningde City (26°85′N, 120°03′E), Fujian Province, China. Three sampling points were designated at each farm; each sampling point was approximately half nautical mile apart. A total of 18 samples were obtained. Each sample contained approximately 100 *P. haitanensis* thalli. The thalli were placed in sterile bags containing seawater from the same sampling location and transported to the laboratory within 8 h in a low-temperature (4°C) box. Parts of the thalli were cleaned to extract metagenomic DNA, and the rest were stored in a freezer at −80°C.

To remove loosely associated microbes and other attachments, 20 thalli from each sample were randomly selected and washed three times with sterilized seawater (Burke et al., 2009). Then, the clean thalli were cut into fragments of approximately 3 mm × 3 mm using sterilized blades and cleaned again with sterilized seawater. These thalli were used as material for metagenomic DNA extraction. All steps were performed on a clean bench. Metagenomic DNA was extracted using the DNeasy PowerSoil Kit (QIAGEN, Germany), according to the manufacturer’s instructions (Yan et al., 2019; Ahmed et al., 2021). The concentration of the extracted DNA was determined using the NanoDrop ND-2000 spectrophotometer (NanoDrop, Wilmington, DE, United States), and the mass was evaluated using a 1% agarose gel. To minimize extraction bias, the three replicates from each sampling point were pooled, resulting in six DNA samples that were denoted as RZ-A, RZ-B, RZ-C, ND-A, ND-B, and ND-C.

### Library Preparation, Sequence Preprocessing, and Assembly

Construction of a metagenomic DNA library and sequencing of paired ends (2 × 150 bp) were performed by Shanghai Persenor according to the standard protocol.^1^ Each sample contained approximately 30 GB of raw reads. The 3′ and 5′ end adapters were removed using Trim Galore v0.5.0,^2^ and reads with length < 20 bp were removed. Paired reads were mapped to the *P. haitanensis* genome (GCA_008729055.1) using Bowtie2 v2.3.2.^3^ To minimize the impact of host contamination, we filtered out read sequences aligned to the *P. haitanensis* genome. The PCR replicates were removed using FastUniq v1.1 (Xu et al., 2012). The contamination rate (%) of clean reads was assessed by remapping the reads to the host genome. Non-pareil v3.30 was used to assess the average coverage of each metagenome (Rodriguez-R et al., 2018). The clean reads of each sample were assembled using SPAdes v3.13.0 (metaspades model, kmers were 21, 33, and 55) (Bankevich et al., 2012). Then, we merged all of the unassembled reads and co-assembled them. Finally, all contigs were moved to a single file for subsequent analysis. The assembly results were statistically analyzed using QUAST v4.5 (Gurevich et al., 2013). Contigs with lengths of less than 1,000 bp were filtered. Finally, to check the assembly effect and quality of the data, clean reads were realigned to contigs.

### Taxonomic Assignment and Metagenomic Binning

To observe the microbial community, Kaiju v1.5.0 (Menzel et al., 2016) was used for taxonomic assignment based on the clean reads of each sample. Non-redundant NCBI BLAST was selected as the taxonomic annotation database. The rarefaction curves for each metagenome were plotted using R language v4.0.1 [vegan package, rarecurve(), step = 2,000] based on the Kaiju results.

Binning was performed using MetaBAT2 v2.12.1 (Kang et al., 2019), MaxBin2 v2.2.4 (-max_iteration 5) (Wu et al., 2016), and CONCOCT (Alneberg et al., 2014). The “bin_refinement” function of MetaWRAP v1.1.8 (Uritskiy et al., 2018) was used to optimize the draft genomes (complete > 50%, contamination < 10%). The dRep v2.5.4 (Olm et al., 2017) was used to remove the redundant MAGs.

Using the Genome Taxonomy Database R89 (GTDB) (Parks et al., 2018), taxonomy was assigned to MAGs using GTDB-Tk v1.1.0 (Chaumeil et al., 2020). The “pipe” and “appraise” functions of SingleM v0.13.2^4^ were used to scan for single copy marker genes in both the MAGs and the sequenced raw reads to estimate the proportion of marker genes recovered in the MAGs. The “genome” function of coverM v0.5.0^5^ was used to evaluate the relative abundance of MAGs in each sample based on read alignments. MAGs with a cumulative abundance > 1% (sum of each MAG abundance in each sample) were included in the heatmap visualized using R language (packages including pheatmap and RColorBrewer), as shown in Supplementary Figure 1.

### Phylogenetic Tree Building and Metabolic Reconstruction of the Metagenome-Assembled Genomes

We filtered out MAGs with quality < 40% (calculated as “Quality = Genome completeness (%) - [5 × Contamination (%)]”). A concatenated marker gene tree was inferred using the 190 MAGs after quality control, together with 231 available IMG/M^6^ *Pyropia* isolated genomes (by 29 October 2020) that were ≥ 50% complete and contained ≤ 10% contamination. For comparison, taxonomy was reassigned to the previously *Pyropia*-associated genomes using the GTDB-R89 database (Supplementary Table 1). Thereafter, the bacterial tree was inferred using GTDB-Tk and embellished using ITOL (Letunic and Bork, 2021). As no archaeal genome was isolated and submitted to public databases before this study, phylogenetic distance (PD) and phylogenetic gain (PG) for the bacterial tree were calculated using GenomeTreeTk v0.0.41^7^ to determine the degree to which the phylogenetic diversity from the current study was added to the tree.

The annotation and annotation functions of EnrichM v0.5.0^8^ were used to annotate MAGs with the Kyoto Encyclopedia of Genes and Genome (KEGG) Orthology database (Kanehisa et al., 2021). Genes that were not annotated were assigned as a “hypothetical protein”. Considering that we obtained incomplete microbial genomes, to assign a pathway/module to MAG, the appropriate KEGG pathway/module needed to be ≥ 60% complete, and all key enzymes must be present (as defined in the literature) (Engelberts et al., 2020). Because the KEGG modules had no auxin biosynthesis module and only a partial cobalamin synthesis module, we created specific modules (Supplementary Note 1). The results of the feature annotation are shown in the phylogenetic tree with KO numbers (Supplementary Figures 2–7).

## Results

### Microbial Community Structures of the Phycosphere

After the quality control step, 209 GB of data was obtained. Approximately 100% of the reads had a base quality greater than 99% (quality score > 20). The rate of clean reads mapped to the host genome was less than 0.1%.

**FIGURE 1:**
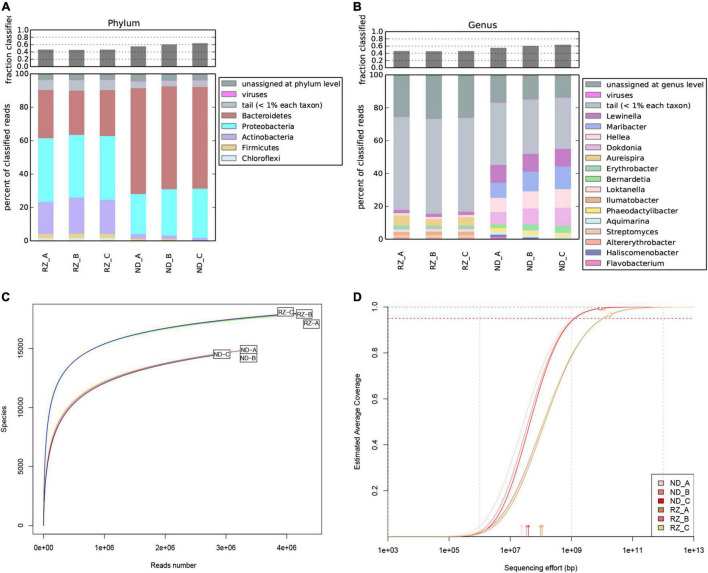
Taxonomic profiling, Rarefaction curves and Non-pareil curves of the *Pyropia haitanensis* microbial dataset. Microbial community structures of *P. haitanensis* at the phylum **(A)** and genus **(B)** level based on non-redundant NCBI BLAST database. Phyla/Genera in different colors are shown in the legend. Categories with relative abundance lower than 1% are merged and defined as “tails”. Reads that are not assigned to any of the above levels are defined as “unassigned at phylum/genus level”. Rarefaction curves **(C)** and Non-pareil curves **(D)** showing estimated average coverage in metagenomic datasets. The empty circles **(D)** indicate the size and estimated current average coverage of the samples. RZ, Rizhao; ND, Ningde.

On average, 47% of the reads were assigned to the taxonomic database. A total of 174 phyla were detected, in which five phyla with relative abundances of over 1% (Figure 1A). A total of 3,433 genera were identified, including 15 genera with relative abundances of more than 1% (Figure 1B). The rarefaction curves (Figure 1C) and non-pareil curves (Figure 1D) indicated that the sequencing data obtained in this study covered more than 95% of the whole microbiome.

Dereplication of the MAGs reduced the total number to 202 (75.05 ± 24.95% completeness, 4.74 ± 4.74% contamination), including 1 archaeal MAG-3 belonging to the phylum Thaumarchaeota (Crenarchaeota as per GTDB) and 201 bacterial MAGs belonging to eight phyla, including Actinobacteriota (12 MAGs), Bacteroidota (100 MAGs), Bdellovibrionota (5 MAGs), Chloroflexota (6 MAGs), Myxococcota (4 MAGs), Patescibacteria (15 MAGs), Proteobacteria (54 MAGs), and Verrucomicrobiota (5 MAGs) (Supplementary Table 2). All dominant taxa with a relative abundance of > 1% were detected in the MAGs. The MAGs contained an average of 3,466 ± 2,747 genes, of which 30.38 ± 20.24% were hypothetical proteins (Supplementary Table 2). Moreover, the analysis of single-copy marker genes presented in the microbial community and MAGs using SingleM showed that 202 MAGs represented 65.2% of the sequenced microbiome. The above results suggested that we obtained a representative dataset of the *Pyropia* microbiome and were able to obtain the metabolic potential of all dominant taxa.

**FIGURE 2:**
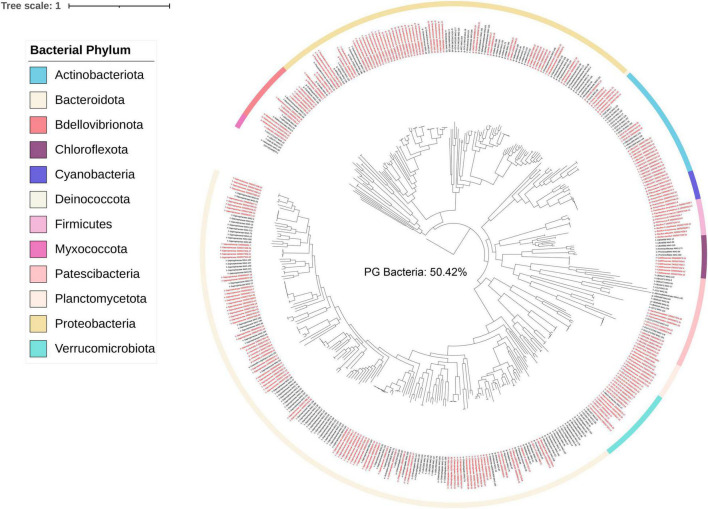
Bacterial phylogenetic tree based on single copy marker proteins (inferred with GTDB). Labels in red are MAGs retrieved from this study. Black labels are *Pyropia* isolated genomes in the IMG/M database. Branch labels display taxonomy at the lowest inferred level. The phylogenetic tree was rooted to “GCA-002453875.1” by GTDB-Tk v1.3.0.

A concatenated genome tree was constructed from 231 *Porphyra/Pyropia*-isolated microbial genomes from the IMG/M database and 190 MAGs after quality control in this study (Figure 2). Although previous genomes were widely distributed across the phylogenetic tree, the 190 MAGs from this study still provided a phylogenetic gain (the additional branch length contributed by a set of taxa) of 50% for the bacteria (Table 1).

**TABLE 1:** Calculated phylogenetic distance (PD) and phylogenetic gain (PG) for the bacterial genome trees, per taxon.

	No. taxa	PD	Percent PD (%)
Full tree	421	75.71	100.00
Outgroup taxa (PD)^a^	231	37.54	49.58
Ingroup taxa (PD)^b^	190	51.07	67.46
Ingroup taxa (PG)	190	38.18	50.42

*^a^Outgroup taxa included previous Pyropia symbiont genomes in the IMG/M database and ^b^ingroup taxa included MAGs retrieved from this study.*

### Autotrophic Carbon Fixation Pathways in the *Pyropia haitanensis* Microbiome

We searched the MAGs for the six known prokaryotic carbon fixation modules in map00720 (Kanehisa et al., 2021), including the Wood-Ljungdahl (WL) pathway (M00377), reductive citric acid (rTCA) cycle (M00173), 3-hydroxypropionate/4-hydroxybutyrate (HP-HB) cycle (M00375), 3-hydroxypropionate (3-HP) cycle (M00376), dicarboxylate/4-hydroxybutyrate (DC-HB) cycle (M00374), and the Calvin Benson Bassham (CBB) cycle (M00165) (Supplementary Table 3). Only the CBB cycle was observed in MAG-99 and MAG-191 (family Rhodobacteraceae) (Supplementary Figure 2). Although the rTCA and the 3-HP cycles had more than 60% module integrity in some MAGs, both lacked the key enzymes ATP-citrate lyases (*aclAB*) and malonyl-CoA reductase (*mcr*), respectively (Zarzycki et al., 2009; Hugler and Sievert, 2011). The other three carbon fixation modules (HP-HB, DC-HB, and WL) were < 60% complete in the MAGs.

### Nitrogen-Transforming Network in the *Pyropia haitanensis* Microbiome

**FIGURE 3:**
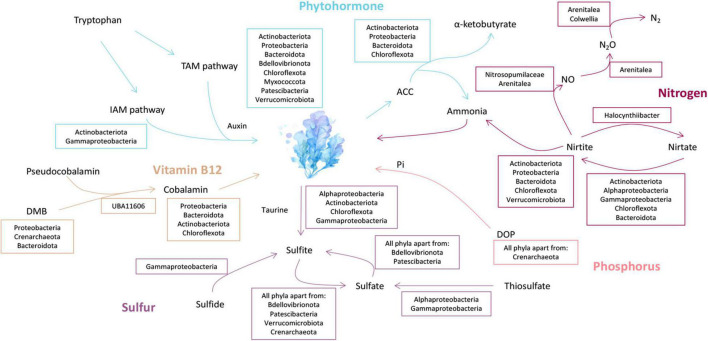
Metabolic reconstruction and the proposed exchange of phytohormone, nitrogen, sulfur, phosphorus, and vitamin B_12_ between *P. haitanensis* symbionts and the host. Blue, phytohormone metabolism; orange, Vitamin B_12_ biosynthesis; purple, sulfur metabolism; pink, phosphorus metabolism; and deep red, nitrogen metabolism. TAM pathway, Tryptamine pathway; IAM pathway, Indole-3-acetamide pathway; DMB, 5,6-dimethylbenzimidazole; DOP, dissolved organic phosphorus; ACC, 1-aminocyclopropane-1-carboxylate. Lineages within the taurine metabolism box contain genes encoding for the taurine transporter (*tauACB*) and taurine dioxygenase (*tauD*). The morphology of *Pyropia haitanensis* is presented in Supplementary Figure 8C.

**FIGURE 4:**
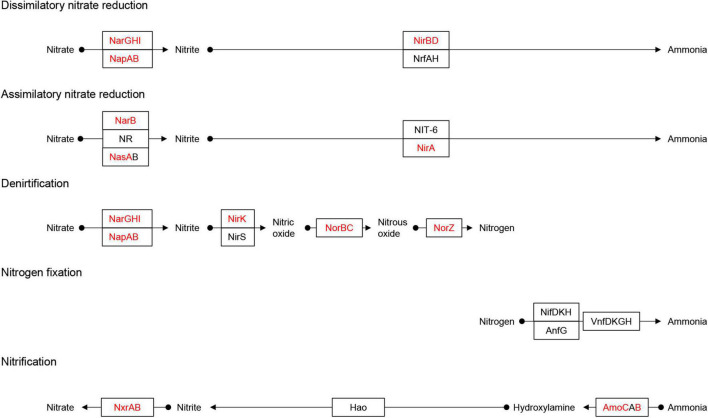
Identification of the Nitrogen metabolism pathway. Adapted from KEGG (https://www.kegg.jp/pathway/map00910). Solid black circles indicate chemical compounds. Squares indicate gene names. The red fonts in the square indicates the names of genes identified in the MAGs, and the black fonts indicates genes that were not identified. The arrow indicates the direction of the reaction pathway.

Nitrate can be reduced to ammonium *via* assimilatory nitrate reduction (ANRA) and dissimilatory nitrate reduction (DNRA) (Devol, 2015). ANRA includes assimilatory nitrate reductases (*nasA* or *narB*) and ferredoxin-nitrite reductase (*nirA*), while DNRA includes nitrate reductases (*narGHI* or *napAB*) and nitrite reductases (*nirBD* or *nrfAH*) (Fiore et al., 2010). The *nasA* and *narB* were identified in Acidimicrobiia (3 MAGs), Alphaproteobacteria (12 MAGs), Gammaproteobacteria (8 MAGs), Anaerolineae (1 MAG), and Bacteroidia (9 MAGs) (Figure 3). Meanwhile, *nirA* was identified in the family DEV007 (2 MAGs). For DNRA, *narGHI or napAB* were identified in the genera *Halocynthiibacter* (MAG-90), *Arenitalea* (MAG-201), and *Colwellia* (MAG-91). Four phyla, Proteobacteria (20 MAGs), Bacteroidetes (8 MAGs), Actinobacteria (3 MAGs), and Chloroflexota (1 MAG), contained *nirBD*. On the other hand, denitrification comprises four steps: nitrate reduction (*narGHI or napAB*), nitrite reduction (*nirK* or *nirS*), nitric oxide reduction (*norBC*), and nitrous oxide reduction (*nosZ*) (Fiore et al., 2010; Figure 4). The *nirK* was identified in MAG-3 (family Nitrosopumilaceae) and MAG-201. Furthermore, MAG-201 had *norBC* and *nosZ*, whereas *nosZ* was also identified in MAG-91. The *nxrAB* encode nitrite oxidoreductase, which catalyzes the nitrite oxidation (Sohaskey and Wayne, 2003). Both genes were identified in MAG-90. Except for the two genes (*amoBC*) of ammonia oxidation identified in MAG-3, KEGG Orthologys (Kos) associated with nitrification (Kanehisa et al., 2021) were absent from other MAGs, as were the KOs involved in nitrogen fixation (Supplementary Figure 3).

### Dissolved Organic Phosphorus Hydrolyzation and Organosulfur Metabolism

One of the most important mechanisms that allow marine organisms to cope with low orthophosphate (Pi) stress is the utilization of Dissolved Organic Phosphorus (DOP) by alkaline phosphatase (AP, *phoABD*) (Lin et al., 2016). The AP genes were identified in 136 MAGs, including Actinobacteria (8 MAGs), Bacteroidetes (86 MAGs), Bdellovibrionota (5 MAGs), Chloroflexota (2 MAGs), Chloroflexota (1 MAG), Myxococcota (1 MAG), Patescibacteria (3 MAGs), Proteobacteria (26 MAGs), and Verrucomicrobiota (5 MAGs) (Figure 3 and Supplementary Figure 4).

**FIGURE 5:**
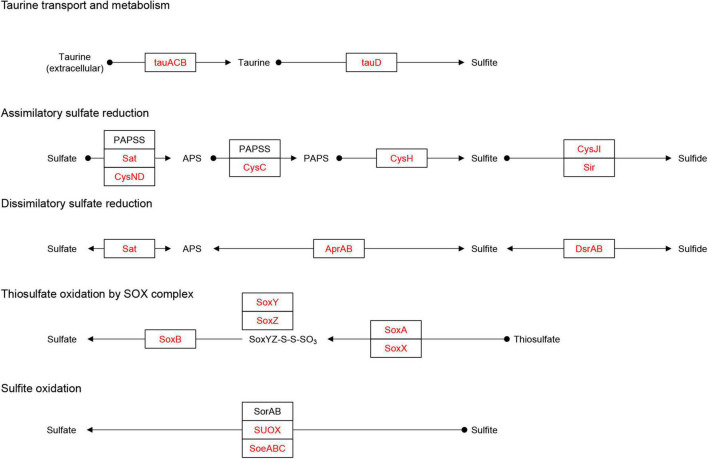
Identification of the sulfur metabolism pathway. Adapted from KEGG (https://www.kegg.jp/pathway/map00920). Solid black circles indicate chemical compounds. Squares indicate gene names. The red fonts in the square indicates the names of genes identified in the MAGs, and the black fonts indicates genes that were not identified. The arrow indicates the direction of the reaction pathway.

Microorganisms can metabolize the host taurine *via* ATP-binding cassette (ABC) transporters (*tauACB*), and taurine is subsequently converted to sulfite *via* taurine dioxygenases (*tauD*) (Rambo et al., 2020; Figure 5). The *tauD* was identified in MAGs belonging to Actinobacteria (8 MAGs), Bacteroidota (1 MAG), Chloroflexota (2 MAGs), Alphaproteobacteria (9 MAGs), and Gammaproteobacteria (4 MAGs), of which *tauACB* transporters were also identified in Actinobacteriota (6 MAGs), Chloroflexota (2 MAGs), Alphaproteobacteria (5 MAGs), and Gammaproteobacteria (3 MAGs) (Figure 3). In addition to the cleavage of taurine, sulfite may also originate from sulfide by dissimilatory sulfite reductase (*dsrAB*) (Engelberts et al., 2020; Figure 5). Both genes were identified in MAG-68 (Gammaproteobacteria) (Supplementary Figure 5). In turn, sulfite can be oxidized to sulfate *via* sulfite dehydrogenase/oxidase (*sorAB/SUOX/soeABC*) (Rambo et al., 2020). The *SUOX/soeABC* were identified in all phyla except Bdellovibrionota, Patescibacteria, Verrucomicrobiota, and Crenarchaeota (Figure 3). The assimilatory sulfate reduction module (M00176), which can reduce sulfate to sulfide (Kanehisa et al., 2021), was found in the phyla Actinobacteria (4 MAGs), Bacteroidetes (47 MAGs), Chloroflexota (5 MAGs), Thaumarchaeota (1 MAG), Myxococcota (3 MAGs), Proteobacteria (9 MAGs), and Verrucomicrobiota (4 MAGs) (Figure 3). The sulfur oxidation system (SOX) is a pathway that includes *soxAB* and *soxXYZ* (Rambo et al., 2020). These genes were found in five Alphaproteobacteria and six Gammaproteobacteria MAGs.

### Metabolic Potential for the Synthesis of Cobalamin

In the present study, potential cobalamin producers in MAGs were identified in different lineages, including the family Cellvibrionaceae (1 MAG), Flavobacteriaceae (1 MAG), Rhizobiaceae (2 MAGs), Rhodobacteraceae (13 MAGs), SZUA-35 (1 MAG), UBA10066 (1 MAG), and UBA6668 (3 MAGs) (Figure 3). Except for MAG-116, MAG-184, and MAG-48, 19 MAGs had the potential to synthesize cobalamin *via* both aerobic and anaerobic routes (Supplementary Figure 6). In addition to *de novo* biosynthesis, cobalamin may also originate from the lower axial ligand conversion of pseudocobalamin (Hoffmann et al., 2000). The *bluB*, which encodes for 5,6-dimethylbenzimidazole synthase (DMB), was present in the families Rhodobacteraceae (6 MAGs), Rhizobiaceae (2 MAGs), Nitrosopumilaceae (1 MAG), and Flavobacteriaceae (1 MAG). Amidohydrolase (*CbiZ*), and cobinamide-phosphate synthase (*CbiB*) can remodel pseudocobalamin into cobalamin by replacing adenine with DMB (Yi et al., 2012). These two genes were only identified in MAG-179 (family UBA11606).

The *btuB*, which encodes for TonB-dependent outer membrane cobalamin receptor and transporter (Noinaj et al., 2010), was identified in the phyla Bacteroidetes (85 MAGs), Bdellovibrionota (3 MAGs), Proteobacteria (19 MAGs), Myxococcota (2 MAGs), and Verrucomicrobiota (4 MAGs) (Supplementary Figure 6). However, few cobalamin biosynthesis genes were found in these MAGs. Moreover, 157 MAGs lacked *metE* (B_12_-independent methionine synthase), while 115 MAGs only included *metH* (B_12_-dependent methionine synthase) (Supplementary Table 3).

### Potential of Metagenome-Assembled Genomes to Produce Auxin and Influence Ethylene Biosynthesis

We searched all the MAGs for the four known 3-indoleacetic acid (IAA) biosynthesis pathways: the indole-3-acetamide (IAM), indole-3-pyruvate (IPyA), tryptamine (TAM), and indole-3-acetonitrile (IAN) pathways (Supplementary Note 1). The metabolic potential of the IAM pathway was present in the families Granulosicoccaceae (3 MAGs) and UBA11606 (2 MAGs) (Figure 3). The TAM pathway was present in 104 MAGs, including the phyla Actinobacteria (8 MAGs), Bacteroidetes (47 MAGs), Bdellovibrionota (1 MAG), Chloroflexota (4 MAGs), Myxococcota (2 MAGs), Patescibacteria (8 MAGs), Proteobacteria (33 MAGs), and Verrucomicrobiota (1 MAG) (Figure 3). However, for the IPyA pathway, the gene encoding the key enzyme (indolepyruvate decarboxylase, *ipdC*) (Duca et al., 2014) was absent in all MAGs (Supplementary Table 3). Furthermore, although some MAGs (7 MAGs in order Chitinophagales) were contained *YUCCA* (encoding the homologs of indole-3-pyruvate monooxygenase) (Mano and Nemoto, 2012), while *Tam1* and *IL4I1* (encoding aminotransferases and L-amino-acid oxidase, respectively) were absent (Supplementary Table 3). The enzymes encoded by *Tam1* and *IL4I1* can transform tryptophan into IPyA (Duca et al., 2014). More than 60% of the genes necessary for carrying out the IAN pathway were found in 11 MAGs; however, indoleacetaldoxime dehydratase (*CYP71A13*) and myrosinase (*E3.2.1.147*) were absent (Supplementary Figure 7). These two genes are related to the conversion of indole-3-acetaldoxime (IAOX) and glucobrassicin to IAN (Zhang et al., 2019).

Microorganisms can promote plant growth indirectly by converting the ethylene precursor 1-aminocyclopropane-1-carboxylate (ACC) to α-ketobutyrate and ammonia by ACC deaminase (*acdS*) (del Carmen Orozco-Mosqueda et al., 2020). The *acdS* was identified in the phyla Actinobacteria (4 MAGs), Bacteroidetes (77 MAGs), Proteobacteria (7 MAGs), and Chloroflexota (1 MAG) (Figure 3), of which 46 MAGs are also potential IAA producers. MAG-144 may contain both TAM and IAM pathways (Supplementary Figure 7).

## Discussion

### Heterotrophic Microorganisms Dominate *Pyropia haitanensis* Microbiota

The products of photosynthesis by plants and algae, such as rhamnose, xylose, and glucose, can provide a carbon source for epiphytic microorganisms (Bruckner et al., 2011; Rigonato et al., 2012; Thapa and Prasanna, 2018). Microorganisms that fix carbon make epiphytic microorganisms less dependent on photosynthetic products and allow them to set a niche for the colonization of other microbes (Rigonato et al., 2012; Thapa and Prasanna, 2018; Flores-Nunez et al., 2020). However, it is still unclear whether all epiphytic microbes are dependent on the carbon provided by *Pyropia*. Here, only two members of Rhodobacteraceae (MAG-99 and MAG-191) fix carbon using the CBB cycle (Supplementary Figure 2), suggesting that epiphytic microorganisms were largely heterotrophic need exogenous carbon to support their life activities. In addition, host-acquired autotrophic carbon from microbial fixation could also contribute to overall high environmental tolerance, particularly to ocean warming (Bell et al., 2018). However, the relative contribution and mechanisms of microbial carbon fixation to marine algal nutrition should be studied further.

### Metabolic Potential of Nutrients

Except for Cyanobacteria, plant cannot fix nitrogen on their own, and the process of nitrogen fixation usually requires the assistance of plant symbiotic microorganisms, such as rhizobia (Cheng, 2008; Kuypers et al., 2018). In our study, no genes associated with nitrogen fixation were found in MAGs, suggesting that the *P. haitanensis* epiphytic microbiomes are unlikely to catalyze the conversion of airborne N_2_ to NH_3_. Within the nitrate reduction reaction, microorganisms can convert nitrate and nitrite to ammonium through ANRA and DNRA (Devol, 2015). A recent study showed the presence of ammonium transporters in *P. yezoensis* (Kakinuma et al., 2017), implying that microbial ammonium may also be trade with the host. Excess nitrite has been shown to have toxic effects on organisms, such as affecting the proton permeability of cell membranes (Vadivelu et al., 2006; Zhou et al., 2010) and inhibiting photosynthesis (Singh et al., 2007). The microbial consumption of nitrite also reduces the risk of excessive nitrite (Vaucheret et al., 1992; Duncanson et al., 1993). Nitrate or nitrite can also be converted to nitrogen oxides (N_2_O and NO) *via* denitrification (Fiore et al., 2010). In addition to being released into the environment, NO is an effective signaling molecule in plant-rhizobacteria interactions, promoting plant growth and development in an auxin-dependent manner (Lamattina et al., 2003; Molina-Favero et al., 2008). N_2_O is a potent greenhouse gas and ozone-depleting substance, and N_2_O emissions can cause atmospheric pollution (Ravishankara et al., 2009). Hence, MAG-91 and MAG-201 may also contribute to the reduction of N_2_O emissions to the environment.

Phosphorus is a central component of nucleic acids and phospholipids and plays a central role in the production of chemical energy (Forlani et al., 2008). However, algae grown in coastal regions may experience Pi starvation because of the rapid consumption of Pi for photosynthesis (Lin et al., 2016). Moreover, anthropogenic N also increases the N:P ratio in receiving waters (Zhang et al., 2007; Zhou et al., 2017), thus limiting the Pi of surrounding organisms. Although there are other forms of phosphorus sources in the ocean, organisms preferentially utilize Pi as they can be taken up directly (Huang et al., 2005; Lin et al., 2016). The major mechanism by which marine phytoplankton and bacteria can convert DOP to bioavailable Pi is the induction of alkaline phosphatase (*phoABD*) (Suzumura et al., 2012; Lin et al., 2019). Here, *phoABD* were identified in 136 MAGs (Supplementary Figure 4). DOP hydrolysis has costly energetic metabolic activity because of the stable C-P bonds (Lin et al., 2016). In terrestrial plants, while both plant and microbial phosphatases are efficient in releasing Pi from soil organic phosphorus, there is evidence that microbial enzymes show a higher efficiency of phosphorus release (Tarafdar et al., 2001). Therefore, it is possible that the “extra” microbial Pi can be assimilated to support host metabolism and growth (Jones, 1972; Cole, 1982; Currie, 1990; Hoppe, 2003).

Taurine, dietary fibers, polyunsaturated fatty acids, and sulfated polysaccharides are enriched in *Pyropia* (Cao et al., 2016; Cho and Rhee, 2020). Our results showed the potential of 16 MAGs to obtain foreign taurine *via* ABC transporters and convert it to sulfite *via* dioxygenases (Supplementary Figure 5). Sulfate produced during dissimilatory sulfate oxidation and dissimilatory thiosulfate oxidation may provide the raw material for assimilatory sulfate reduction for cysteine (Takahashi et al., 2011) and other cell material biosynthesis. Similar to the nitrogen cycle, genes required for certain reactions in the sulfur cycle have been identified only in specific microbial lineages, implies that they may play prominent roles in nitrogen/sulfur cycling within the *Pyropia* phycosphere.

### Cobalamin and Phytohormone Metabolism

In many organisms with *metH*, vitamin B_12_ usually acts as an enzyme cofactor participating in catalyzing primary biochemical reactions (Sanudo-Wilhelmy et al., 2014), such as amino acid (Banerjee and Matthews, 1990) and DNA synthesis (Blakley and Barker, 1964). Currently, cobalamin is thought to be synthesized by a relatively small set of prokaryotes, and algae may obtain cobalamin from these associated microbes (Giovannoni, 2012; Lynch and Neufeld, 2015). Our previous study revealed six potential cobalamin producers, all of which belong to Alphaproteobacteria (Wang et al., 2021). Here, 22 MAGs in five phyla were considered potential producers of cobalamin through aerobic or anaerobic pathway (Supplementary Figure 6). This result suggested that vitamin B_12_ producers were not restricted to a particular microbial lineage. Another source of cobalamin is the substitution of the adenine lower axial ligand of pseudocobalamin by DMB (Helliwell et al., 2016). Pseudocobalamin is a vitamer that can be synthesized in cyanobacteria (Hoffmann et al., 2000). Furthermore, amidohydrolase (*cbiZ*) and cobinamide-phosphate synthase (*cbiB*) can replace the adenine lower axial ligand with DMB (Yi et al., 2012). In this study, 10 MAGs were identified to have DMB synthesis genes, and one MAG (MAG-179) was identified to have remodeled pseudocobalamin genes. These MAGs may play a key role in maximizing the effect of cobalamin production (Lu et al., 2020). In terms of potential cobalamin consumption, we found 113 MAGs with the TonB-dependent cobalamin transporter gene (*btuB*) and 115 MAGs with the B_12_-dependent methionine synthase gene (*metH*). The MAGs with *btuB* had low complete of the cobalamin synthesis pathway (Supplementary Figure 6). This phenomenon was also presented in soil cobalamin research (Lu et al., 2020); our study was more specific by presenting at the individual level. Both *btuB* and *metH* were identified in more than half of the MAGs, suggesting that cobalamin producers play important roles in the microbial community (Banerjee and Matthews, 1990; Lu et al., 2020).

Auxin is one of the important plant hormones that influence plant growth and development (Vanneste and Friml, 2009; Bidon et al., 2020). In the rhizosphere, the potential to synthesize auxin is considered the main characteristic of plant growth-promoting rhizobacteria (PGPR) (Spaepen et al., 2007). A previous study showed that co-culture with IAA producers can positively affect the growth of *P. yezoensis* (Matsuda et al., 2018). Furthermore, bacterial IAA is also reported to regulate microbial physiology, including overcoming stress, regulating cellular processes, and providing a carbon and nitrogen source (Bianco et al., 2006; Tromas and Perrot-Rechenmann, 2010; Van Puyvelde et al., 2011). Here, the IAM and TAM pathways were present in 109 MAGs, including diverse microbial lineages (Supplementary Figure 7). Both pathways are also widely distributed in rhizobacteria (Zhang et al., 2019). The IPyA pathway was usually discovered in plant growth-promoting rhizobacteria (Fu et al., 2012); however, the key gene (*ipdC*) was absent in the MAGs (Supplementary Figure 7). Notably, although the IPyA and IAN pathways lack genes encoding key enzymes, they both have a high degree of integrity in some MAGs (Supplementary Table 3). Therefore, it does not exclude the possibility that bacteria can synthesize IAA from intermediates of other microbial lineages (Zhang et al., 2019), even of host (Amin et al., 2015).

Ethylene is associated with plant senescence, abscission, flower development, and pathogen defense signaling (Lin et al., 2009; Van de Poel et al., 2015). Ethylene biosynthesis is induced under diverse stress conditions, thereby inhibiting plant growth (Abeles et al., 2012). In this process, ACC is an important intermediate (Lin et al., 2009). A previous report showed that exogenous addition of ACC can increase ethylene production and promote the formation of spermatia and zygotospores in *P. yezoensis* (Uji et al., 2020). In the rhizosphere, microbial ACC deaminase can metabolize ACC exuded by plants, resulting in a reduction or stalling of ethylene biosynthesis inside the host owing to a decrease in the precursor (Fu et al., 2012), and it is also another feature of PGPR (Wang et al., 2007; Bulgarelli et al., 2013). In this study, the 89 MAGs possessed the ACC deaminase gene, of which 46 MAGs also possessed IAA synthesis potential (Supplementary Figure 7). These MAGs with “dual identity” may be advantageous for future screening of beneficial bacteria (Lugtenberg and Kamilova, 2009).

### Metabolic Potential of Archaea

Although archaea are extremely widespread in the biosphere (Vaucheret et al., 1992), their potential role in macroalgae is rarely mentioned (Trias et al., 2012; Karray et al., 2017; Ghosh et al., 2019), especially in *Pyropia*. In this study, we obtained an archaeal draft genome (MAG-3) belonging to the Nitrosopumilaceae family. Our result filled a gap in the understanding of the archaeal genome in the epiphytic microorganisms of *Pyropia*. Further functional analysis of MAG-3 revealed its potential role in MAG-3 ammonia oxidation (Supplementary Figure 3), nitrite reduction, DMB biosynthesis, and assimilatory sulfate reduction (Supplementary Figures 5, 6). However, there may be some other functional features that have not been identified owing to the incomplete genome.

### Different Metabolic Potentials Between Two Sampling Sites

We also evaluated the differences between microbial communities though the abundance of MAGs (Supplementary Figure 9). Regarding inorganic salt metabolism, the relative abundance of MAGs with DOP metabolic potential was higher in the ND group (RZ vs. ND: 56.6% vs. 78.31%); in contrast, the relative abundance of MAGs with sulfur and nitrogen metabolic potential was higher in the RZ group (S: 61.58% vs. 52.93%; N: 17.16% vs. 15.52%). In terms of phytohormone anabolism, the relative abundance of MAGs with IAA synthesis potential exceeded 60% in both groups (RZ vs. ND: 77.58% vs. 61.76%); the relative abundance of MAGs with metabolic ACC potential was higher in the ND group (35.78% vs. 53.05%). Both environment and algae are capable of influencing the community structure of epiphytic microorganisms, so we speculate that the reason for these differences may be the result of a combination of environmental and algal effects (Yan et al., 2019; Wang et al., 2020; Ahmed et al., 2021).

It should be emphasized that our results and conclusions were based on bioinformatics methods. Although we recovered 65.2% of the sequencing community, the remaining 35.8% of unbinned sequences may contain functions that we have not characterized. In addition, bioinformatics-based predictions may include incomplete metabolic pathways because of incomplete genome analysis. We believe that future studies aimed at understanding *Pyropia* as a holobiont should also move toward the retrieval of host genes and genomes so that the metabolic potential of *Pyropia* and its epiphytic microorganisms can be assessed for metabolic complementarity.

## Data Availability Statement

The datasets presented in this study can be found in online repositories. The names of the repository/repositories and accession number(s) can be found below: https://www.ncbi.nlm.nih.gov/, PRJNA726403.

## Author Contributions

JW, ZM, and YM designed the experiments and prepared the manuscript. JW and XT collected the materials. JW did the experiments and bioinformatics analysis. All authors were involved in revision of the manuscript and approved its final version.

## Conflict of Interest

The authors declare that the research was conducted in the absence of any commercial or financial relationships that could be construed as a potential conflict of interest.

## Publisher’s Note

All claims expressed in this article are solely those of the authors and do not necessarily represent those of their affiliated organizations, or those of the publisher, the editors and the reviewers. Any product that may be evaluated in this article, or claim that may be made by its manufacturer, is not guaranteed or endorsed by the publisher.

## Footnotes

1
http://www.illumina.com/

2
https://github.com/FelixKrueger/TrimGalore

3
https://github.com/BenLangmead/bowtie2

4
https://github.com/wwood/singlem

5
https://github.com/wwood/CoverM

6
https://img.jgi.doe.gov/cgi-bin/m/main.cgi

7
https://github.com/dparks1134/GenomeTreeTK

8
https://github.com/geronimp/enrichM

## Supplementary Material

Supplementary Figure 1 Relative abundance of the dominant MAGs (>1% abundance across the data). “Unmapped” represents reads that could not be mapped to any of the 202 MAGs. RZ, Rizhao; ND, Ningde.

Supplementary Figure 2 KO absence/presence in the MAGs retrieved from *Pyropia haitanensis* that are involved in six autotrophic carbon fixation pathways found in marine prokaryotes, from left to right: Wood-Ljungdahl pathway, reductive citric acid cycle, hydroxypropionate-hydroxybutyrate cycle, 3-hydroxypropionate bi-cycle, dicarboxylate-hydroxyburyrate cycle, and the Calvin-Benson-Bassham cycle. Corresponding KEGG modules are displayed on the left of the figure. Similar colored squares indicate KOs involved in the same step of the module as defined by KEGG. Blue colored lineages have metabolic potential for the Calvin-Benson-Bassham cycle. Branch labels display taxonomy at the lowest inferred level. Bacterial tree was rooted to GCA_002453875.1. The KEGG module was retrieved from https://www.genome.jp/on December 7, 2020.

Supplementary Figure 3 KO absence/presence in the MAGs retrieved from *Pyropia haitanensis* that are involved in nitrogen fixation, nitrification, denitrification, assimilatory nitrate reduction, and dissimilatory nitrate reduction. Corresponding KEGG modules are displayed on the left of the figure. Similar colored squares indicate KOs involved in the same step of the module as defined by KEGG. Branch labels display taxonomy at the lowest inferred level. Bacterial tree was rooted to GCA_002453875.1. KEGG modules were retrieved from https://www.genome.jp/on December 7, 2020.

Supplementary Figure 4 KO absence/presence in the MAGs retrieved from *Pyropia haitanensis* that are involved in taurine transport, sulfide and sulfite oxidation, and sulfate reduction. Corresponding KEGG modules are displayed on the left of the figure. Similar colored squares indicate KOs involved in the same step of the module as defined by KEGG. Branch labels display taxonomy at the lowest inferred level. Bacterial tree was rooted to GCA_002453875.1. KEGG modules were retrieved from https://www.genome.jp/on December 7, 2020.

Supplementary Figure 5 KO absence/presence in the MAGs retrieved from *Pyropia haitanensis* that are involved in hydrolyzing dissolved organic phosphorus (DOP). Similar colored squares indicate KOs involved in the same step of the module as defined by KEGG. Blue colored lineages have the metabolic potential to hydrolyze DOP. Branch labels display taxonomy at the lowest inferred level. Bacterial tree was rooted to GCA_002453875.1.

Supplementary Figure 6 KO absence/presence in MAGs retrieved from *Pyropia haitanensis* that are involved in indole-3-acetic acid synthesis and ACC deaminase (K01505). Corresponding KEGG modules are displayed on the left of the figure. Similar colored squares indicate KOs involved in the same step of the module as defined by KEGG. Blue colored lineages have the metabolic potential to produce IAA by the TAM pathway. Bold lineages have the metabolic potential to produce IAA by the IAM pathway. Italic lineages have the metabolic potential to produce ACC deaminase. Branch labels display taxonomy at the lowest inferred level. Bacterial tree was rooted to GCA_002453875.1. KEGG modules were retrieved from https://www.genome.jp/on December 7, 2020.

Supplementary Figure 7 KO absence/presence in MAGs retrieved from *Pyropia haitanensis* that are involved in cobalamin (vitamin B_12_) metabolism. Blue colored lineages can metabolize cobalamin through either one of the two pathways. Bold lineages have the metabolic potential to produce 5,6-dimethylbenzimidazole (DMB). Red colored lineages can remodel cobalamin-like compounds (such as pseudocobalamin) into cobalamin. Branch labels display taxonomy at the lowest inferred level. Bacterial tree was rooted to GCA_002453875.1.

Supplementary Figure 8 The ongrowing technique and morphology of *Pyropia haitanensis*. The fixed nets are hung between poles **(A,B)**. **(C)** Is morphology of *Pyropia haitanensis*.

Supplementary Figure 9 Evaluation of the differences between microbial communities. The *X*-axis shows the relative abundance of MAGs with metabolic potential. ACC, 1-aminocyclopropane-1-carboxylate; DOP, dissolved organic phosphorus; IAM, Indole-3-acetamide pathway; N, Nitrogen metabolism; S, Sulfur metabolism; TAM, Tryptamine pathway; RZ, Rizhao; ND, Ningde.

Supplementary Table 1 Taxonomy and statistics of the IMG/M database with *Pyropia* symbiont genomes included in the concatenated marker gene tree. The GTDB R89 taxonomy is presented.

Supplementary Table 2 Taxonomy and statistics of 202 MAGs retrieved from this study. The GTDB R89 taxonomy is presented.

Supplementary Table 3 KO count for each of the 202 MAGs retrieved in this study. The GTDB R89 taxonomy is presented. KOs are involved in the described metabolic pathways (i.e., carbon fixation, nitrogen, sulfur, phytohormone, and vitamin B_12_).

Supplementary Note 1 Auxin biosynthesis modules and cobalamin synthesis modules.
