# Supplementary figures and images for: Metagenome-Assembled Genomes From Pyropia haitanensis Microbiome Provide Insights Into the Potential Metabolic Functions to the Seaweed
Source: Front Microbiol. 2022 Mar 23;13:857901. doi: 10.3389/fmicb.2022.857901 (PMC8984609; doi:10.3389/fmicb.2022.857901)

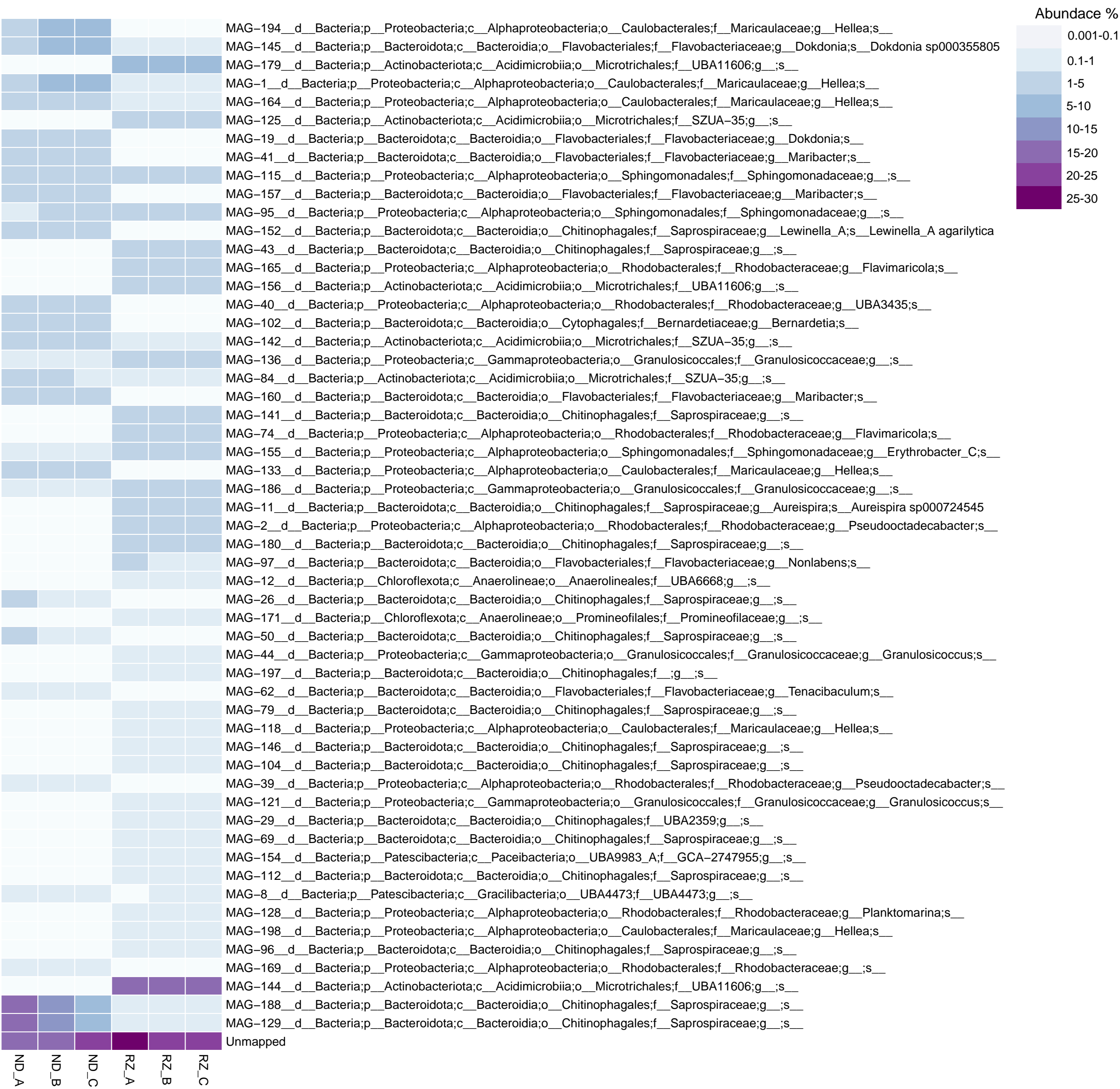

Supplement: Supplementary Figure 1 — Relative abundance of the dominant MAGs (>1% abundance across the data). “Unmapped” represents reads that could not be mapped to any of the 202 MAGs. RZ, Rizhao; ND, Ningde. [file Image_1.PDF]

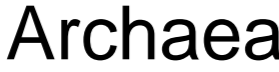

Supplement: Supplementary Figure 2 — KO absence/presence in the MAGs retrieved from Pyropia haitanensis that are involved in six autotrophic carbon fixation pathways found in marine prokaryotes, from left to right: Wood-Ljungdahl pathway, reductive citric acid cycle, hydroxypropionate-hydroxybutyrate cycle, 3-hydroxypropionate bi-cycle, dicarboxylate-hydroxyburyrate cycle, and the Calvin-Benson-Bassham cycle. Corresponding KEGG modules are displayed on the left of the figure. Similar colored squares indicate KOs involved in the same step of the module as defined by KEGG. Blue colored lineages have metabolic potential for the Calvin-Benson-Bassham cycle. Branch labels display taxonomy at the lowest inferred level. Bacterial tree was rooted to GCA_002453875.1. The KEGG module was retrieved from https://www.genome.jp/on December 7, 2020. [file Image_2.PDF]

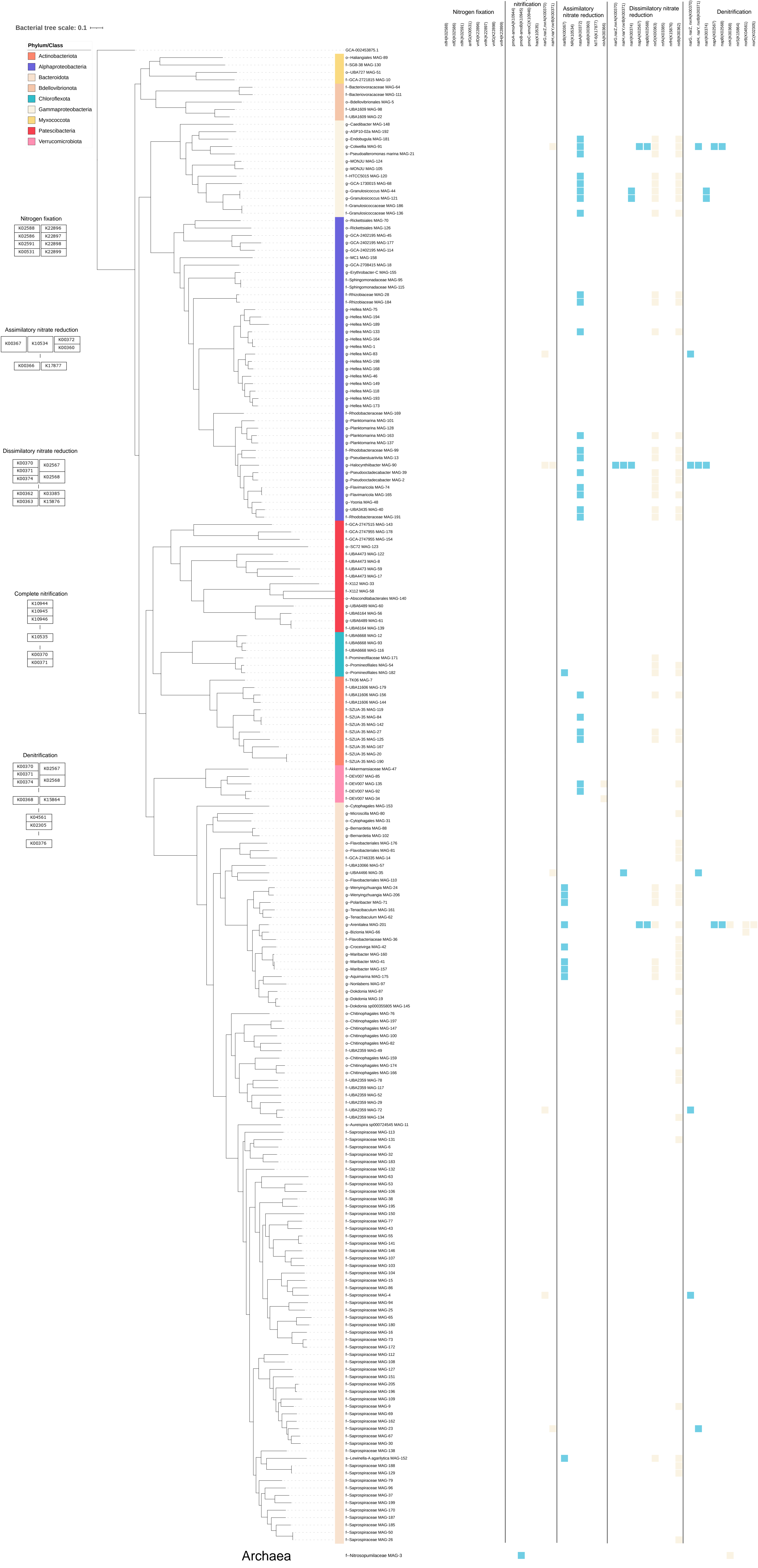

Supplement: Supplementary Figure 3 — KO absence/presence in the MAGs retrieved from Pyropia haitanensis that are involved in nitrogen fixation, nitrification, denitrification, assimilatory nitrate reduction, and dissimilatory nitrate reduction. Corresponding KEGG modules are displayed on the left of the figure. Similar colored squares indicate KOs involved in the same step of the module as defined by KEGG. Branch labels display taxonomy at the lowest inferred level. Bacterial tree was rooted to GCA_002453875.1. KEGG modules were retrieved from https://www.genome.jp/on December 7, 2020. [file Image_3.PDF]

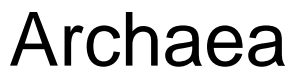

Supplement: Supplementary Figure 5 — KO absence/presence in the MAGs retrieved from Pyropia haitanensis that are involved in hydrolyzing dissolved organic phosphorus (DOP). Similar colored squares indicate KOs involved in the same step of the module as defined by KEGG. Blue colored lineages have the metabolic potential to hydrolyze DOP. Branch labels display taxonomy at the lowest inferred level. Bacterial tree was rooted to GCA_002453875.1. [file Image_5.PDF]

Phylogenetic tree scale: 0.1

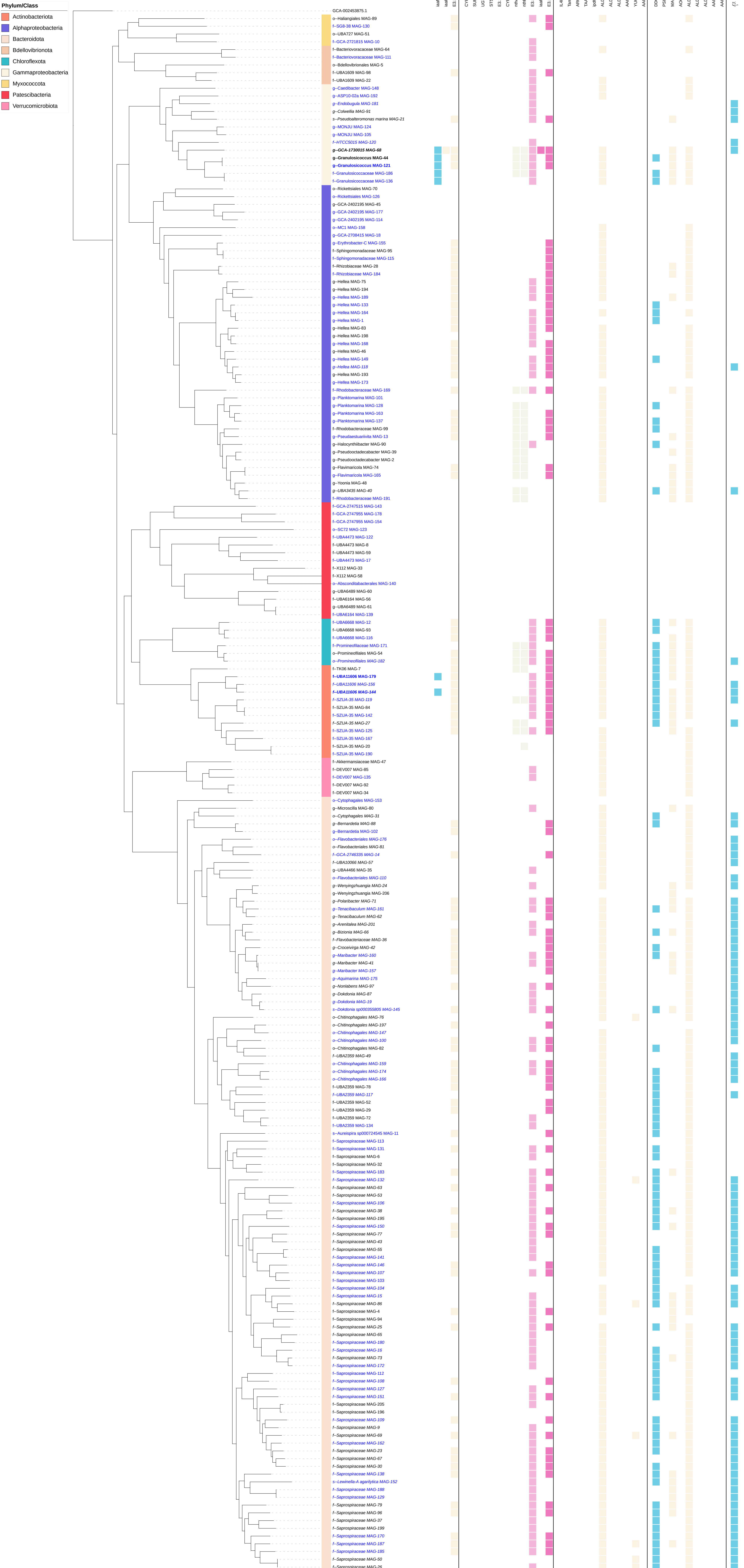

# Archaea

Supplement: Supplementary Figure 7 — KO absence/presence in MAGs retrieved from Pyropia haitanensis that are involved in cobalamin (vitamin B12) metabolism. Blue colored lineages can metabolize cobalamin through either one of the two pathways. Bold lineages have the metabolic potential to produce 5,6-dimethylbenzimidazole (DMB). Red colored lineages can remodel cobalamin-like compounds (such as pseudocobalamin) into cobalamin. Branch labels display taxonomy at the lowest inferred level. Bacterial tree was rooted to GCA_002453875.1. [file Image_7.PDF]

A

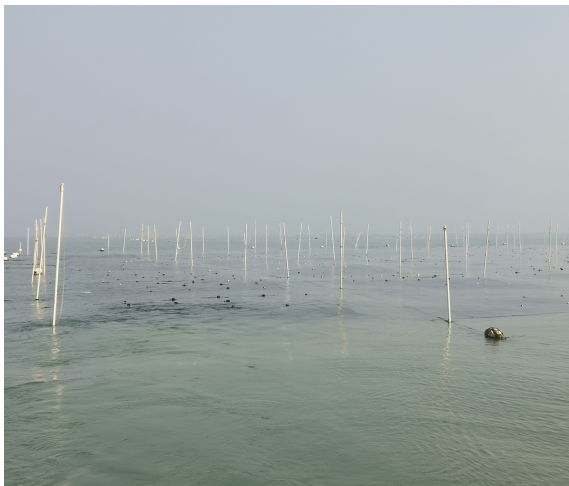

B

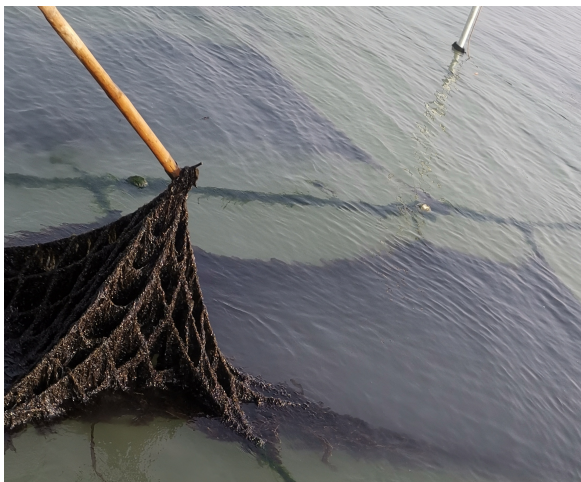

C

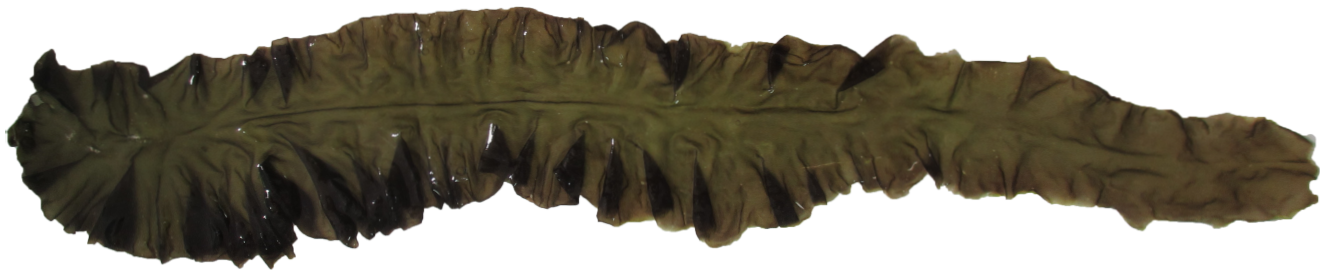

Supplement: Supplementary Figure 8 — The ongrowing technique and morphology of Pyropia haitanensis. The fixed nets are hung between poles (A,B). (C) Is morphology of Pyropia haitanensis. [file Image_8.PDF]

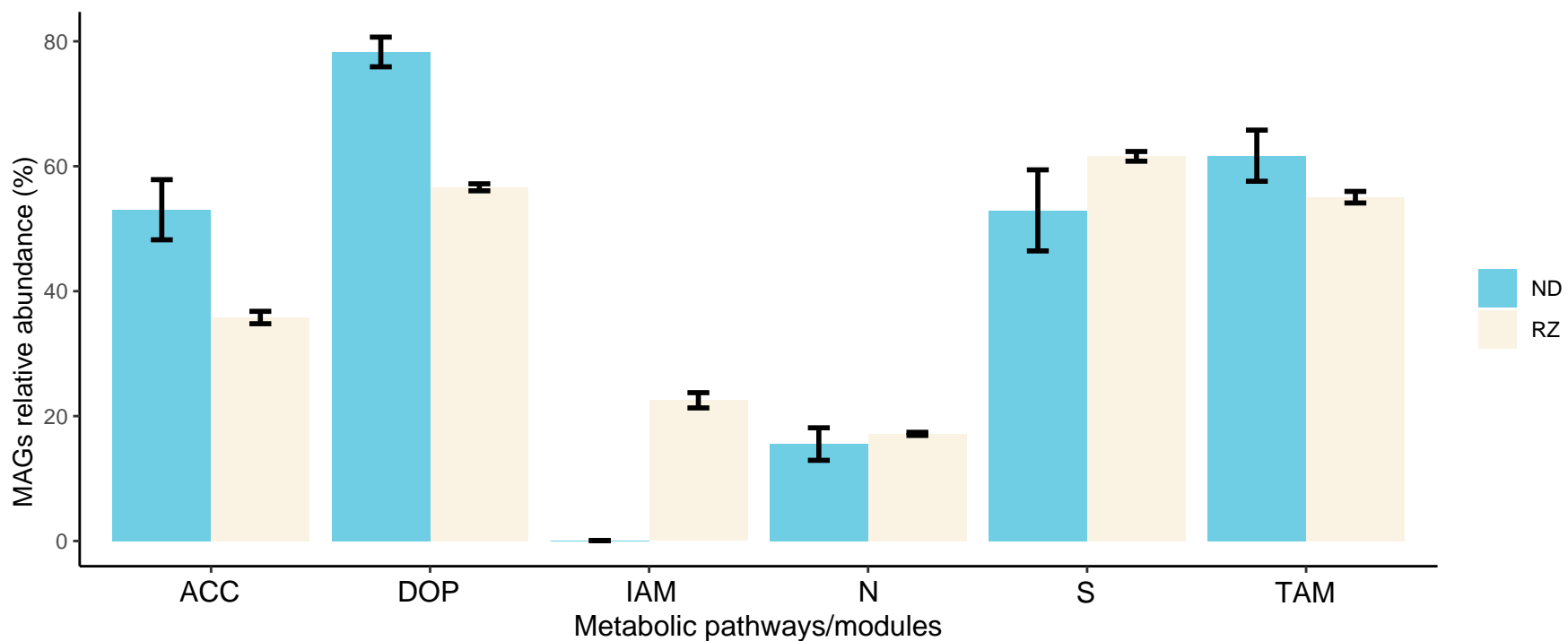

Supplement: Supplementary Figure 9 — Evaluation of the differences between microbial communities. The X-axis shows the relative abundance of MAGs with metabolic potential. ACC, 1-aminocyclopropane-1-carboxylate; DOP, dissolved organic phosphorus; IAM, Indole-3-acetamide pathway; N, Nitrogen metabolism; S, Sulfur metabolism; TAM, Tryptamine pathway; RZ, Rizhao; ND, Ningde. [file Image_9.PDF]
