# Supplementary material for: Metagenome-Assembled Genomes From Pyropia haitanensis Microbiome Provide Insights Into the Potential Metabolic Functions to the Seaweed
Source: Front Microbiol. 2022 Mar 23;13:857901. doi: 10.3389/fmicb.2022.857901 (PMC8984609; doi:10.3389/fmicb.2022.857901)
Supplement: Supplementary Figure 4 — KO absence/presence in the MAGs retrieved from Pyropia haitanensis that are involved in taurine transport, sulfide and sulfite oxidation, and sulfate reduction. Corresponding KEGG modules are displayed on the left of the figure. Similar colored squares indicate KOs involved in the same step of the module as defined by KEGG. Branch labels display taxonomy at the lowest inferred level. Bacterial tree was rooted to GCA_002453875.1. KEGG modules were retrieved from https://www.genome.jp/on December 7, 2020. [file Image_4.PDF]

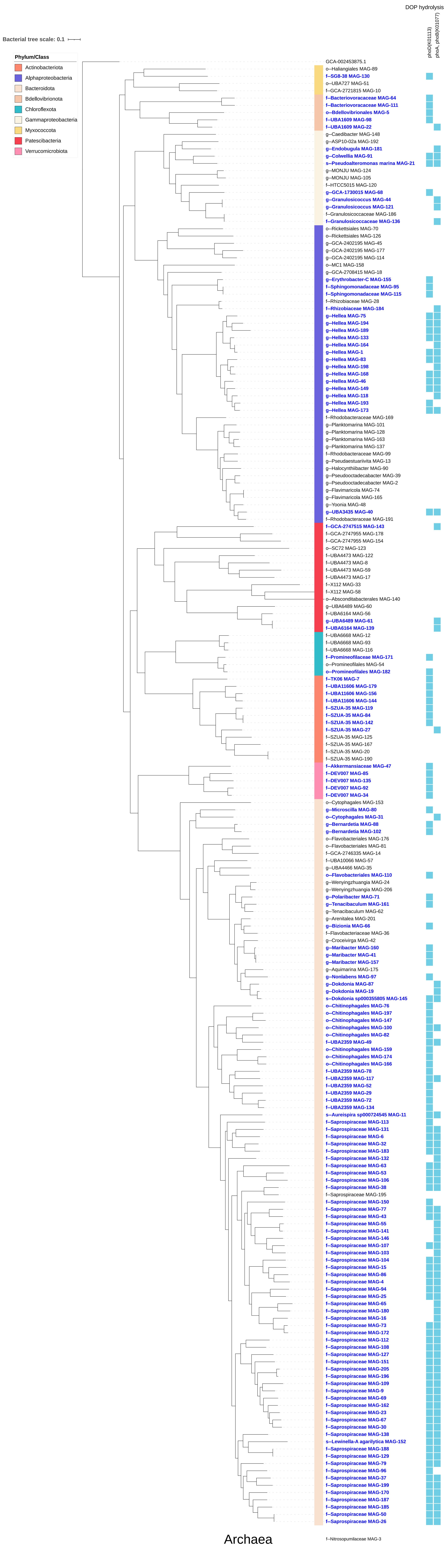
DOP hydrolysis
phoD(K01113)
phoA, phoB(K01077)
Bacterial tree scale: 0.1
Phylum/Class
Actinobacteriota
Alphaproteobacteria
Bacteroidota
Bdellovibrionota
Chloroflexota
Gammaproteobacteria
Myxococcota
Patescibacteria
Verrucomicrobiota
GCA-002453875.1
o--Haliangiales MAG-89
f--SG8-38 MAG-130
o--UBA727 MAG-51
f--GCA-2721815 MAG-10
f--Bacteriovoracaceae MAG-64
f--Bacteriovoracaceae MAG-111
o--Bdellovibrionales MAG-5
f--UBA1609 MAG-98
f--UBA1609 MAG-22
g--Caedibacter MAG-148
g--ASP10-02a MAG-192
g--Endobugula MAG-181
g--Colwellia MAG-91
s--Pseudoalteromonas marina MAG-21
g--MONJU MAG-124
g--MONJU MAG-105
f--HTCC5015 MAG-120
g--GCA-1730015 MAG-68
g--Granulosicoccus MAG-44
g--Granulosicoccus MAG-121
f--Granulosicoccaceae MAG-186
f--Granulosicoccaceae MAG-136
o--Rickettsiales MAG-70
o--Rickettsiales MAG-126
g--GCA-2402195 MAG-45
g--GCA-2402195 MAG-177
g--GCA-2402195 MAG-114
o--MC1 MAG-158
g--GCA-2708415 MAG-18
g--Erythrobacter-C MAG-155
f--Sphingomonadaceae MAG-95
f--Sphingomonadaceae MAG-115
f--Rhizobiaceae MAG-28
f--Rhizobiaceae MAG-184
g--Hellea MAG-75
g--Hellea MAG-194
g--Hellea MAG-189
g--Hellea MAG-133
g--Hellea MAG-164
g--Hellea MAG-1
g--Hellea MAG-83
g--Hellea MAG-198
g--Hellea MAG-168
g--Hellea MAG-46
g--Hellea MAG-149
g--Hellea MAG-118
g--Hellea MAG-193
g--Hellea MAG-173
f--Rhodobacteraceae MAG-169
g--Planktomarina MAG-101
g--Planktomarina MAG-128
g--Planktomarina MAG-163
g--Planktomarina MAG-137
f--Rhodobacteraceae MAG-99
g--Pseudaestuariivita MAG-13
g--Halocynthiibacter MAG-90
g--Pseudooctadecabacter MAG-39
g--Pseudooctadecabacter MAG-2
g--Flavimaricola MAG-74
g--Flavimaricola MAG-165
g--Yoonia MAG-48
g--UBA3435 MAG-40
f--Rhodobacteraceae MAG-191
f--GCA-2747515 MAG-143
f--GCA-2747955 MAG-178
f--GCA-2747955 MAG-154
o--SC72 MAG-123
f--UBA4473 MAG-122
f--UBA4473 MAG-8
f--UBA4473 MAG-59
f--UBA4473 MAG-17
f--X112 MAG-33
f--X112 MAG-58
o--Absconditabacterales MAG-140
g--UBA6489 MAG-60
f--UBA6164 MAG-56
g--UBA6489 MAG-61
f--UBA6164 MAG-139
f--UBA6668 MAG-12
f--UBA6668 MAG-93
f--UBA6668 MAG-116
f--Promineofilaceae MAG-171
o--Promineofilales MAG-54
o--Promineofilales MAG-182
f--TK06 MAG-7
f--UBA11606 MAG-179
f--UBA11606 MAG-156
f--UBA11606 MAG-144
f--SZUA-35 MAG-119
f--SZUA-35 MAG-84
f--SZUA-35 MAG-142
f--SZUA-35 MAG-27
f--SZUA-35 MAG-125
f--SZUA-35 MAG-167
f--SZUA-35 MAG-20
f--SZUA-35 MAG-190
f--Akkermansiaceae MAG-47
f--DEV007 MAG-85
f--DEV007 MAG-135
f--DEV007 MAG-92
f--DEV007 MAG-34
o--Cytophagales MAG-153
g--Microscilla MAG-80
o--Cytophagales MAG-31
g--Bernardetia MAG-88
g--Bernardetia MAG-102
o--Flavobacteriales MAG-176
o--Flavobacteriales MAG-81
f--GCA-2746335 MAG-14
f--UBA10066 MAG-57
g--UBA4466 MAG-35
o--Flavobacteriales MAG-110
g--Wenyingzhuangia MAG-24
g--Wenyingzhuangia MAG-206
g--Polaribacter MAG-71
g--Tenacibaculum MAG-161
g--Tenacibaculum MAG-62
g--Arenitalea MAG-201
g--Bizionia MAG-66
f--Flavobacteriaceae MAG-36
g--Croceivirga MAG-42
g--Maribacter MAG-160
g--Maribacter MAG-41
g--Maribacter MAG-157
g--Aquimarina MAG-175
g--Nonlabens MAG-97
g--Dokdonia MAG-87
g--Dokdonia MAG-19
s--Dokdonia sp000355805 MAG-145
o--Chitinophagales MAG-76
o--Chitinophagales MAG-197
o--Chitinophagales MAG-147
o--Chitinophagales MAG-100
o--Chitinophagales MAG-82
f--UBA2359 MAG-49
o--Chitinophagales MAG-159
o--Chitinophagales MAG-174
o--Chitinophagales MAG-166
f--UBA2359 MAG-78
f--UBA2359 MAG-117
f--UBA2359 MAG-52
f--UBA2359 MAG-29
f--UBA2359 MAG-72
f--UBA2359 MAG-134
s--Aureispira sp000724545 MAG-11
f--Saprospiraceae MAG-113
f--Saprospiraceae MAG-131
f--Saprospiraceae MAG-6
f--Saprospiraceae MAG-32
f--Saprospiraceae MAG-183
f--Saprospiraceae MAG-132
f--Saprospiraceae MAG-63
f--Saprospiraceae MAG-53
f--Saprospiraceae MAG-106
f--Saprospiraceae MAG-38
f--Saprospiraceae MAG-195
f--Saprospiraceae MAG-150
f--Saprospiraceae MAG-77
f--Saprospiraceae MAG-43
f--Saprospiraceae MAG-55
f--Saprospiraceae MAG-141
f--Saprospiraceae MAG-146
f--Saprospiraceae MAG-107
f--Saprospiraceae MAG-103
f--Saprospiraceae MAG-104
f--Saprospiraceae MAG-15
f--Saprospiraceae MAG-86
f--Saprospiraceae MAG-4
f--Saprospiraceae MAG-94
f--Saprospiraceae MAG-25
f--Saprospiraceae MAG-65
f--Saprospiraceae MAG-180
f--Saprospiraceae MAG-16
f--Saprospiraceae MAG-73
f--Saprospiraceae MAG-172
f--Saprospiraceae MAG-112
f--Saprospiraceae MAG-108
f--Saprospiraceae MAG-127
f--Saprospiraceae MAG-151
f--Saprospiraceae MAG-205
f--Saprospiraceae MAG-196
f--Saprospiraceae MAG-109
f--Saprospiraceae MAG-9
f--Saprospiraceae MAG-69
f--Saprospiraceae MAG-162
f--Saprospiraceae MAG-23
f--Saprospiraceae MAG-67
f--Saprospiraceae MAG-30
f--Saprospiraceae MAG-138
s--Lewinella-A agarilytica MAG-152
f--Saprospiraceae MAG-188
f--Saprospiraceae MAG-129
f--Saprospiraceae MAG-79
f--Saprospiraceae MAG-96
f--Saprospiraceae MAG-37
f--Saprospiraceae MAG-199
f--Saprospiraceae MAG-170
f--Saprospiraceae MAG-187
f--Saprospiraceae MAG-185
f--Saprospiraceae MAG-50
f--Saprospiraceae MAG-26
Archaea
f--Nitrosopumilaceae MAG-3
