# Supplementary material for: Metagenome-Assembled Genomes From Pyropia haitanensis Microbiome Provide Insights Into the Potential Metabolic Functions to the Seaweed
Source: Front Microbiol. 2022 Mar 23;13:857901. doi: 10.3389/fmicb.2022.857901 (PMC8984609; doi:10.3389/fmicb.2022.857901)
Supplement: Supplementary Note 1 — Auxin biosynthesis modules and cobalamin synthesis modules. [file Data_Sheet_1.DOCX]

***Redefinition of cobalamin module***

The cobalamin module derived from KEGG was redefined to include enzymes upstream of Cob(I)yrinate a,c, diamide and downstream of precorrin 2, included aerobic or anaerobic pathways.(1,2).

Redefined module for cobalamin biosynthesis through precorrin (aerobic):

(K03394,K13540), K02229, (K05934,K13540,K13541), K05936, K02228, K05895, K00595, K06042, K02224, (K02230,K09882,K09883), K13786, (K00798,K19221), K02232, (K02225,K02227), K02231, (K02231,K19712), K00768, (K02226,K22316), K02233

Redefined module for cobalamin biosynthesis through co-precorrin (anaerobic):

(K02302,K02304), (K02190,K03795,K22011), (K03394,K13540), (K05934,K13540,K13541)/K21479, K05936, (K02189,K13541), K02188, K05895, (K02191,K00595), (K03399,K00595), K06042, K02224, K13786, (K00798,K19221), K02232, (K02225,K02227), K02231, (K02231,K19712), K00768, (K02226,K22316), K02233

***Redefinition of IAA pathway***

Bacterial IAA synthesis mainly uses tryptophan as the precursor via four different pathways, classified according to their intermediates: indole-3-acetamide (IAM), indole-3-pyruvate, indole-3-acetonitrile (IAN), and tryptamine(TAM) (3,4,5,6).

For IAN pathway, the first step is to convert Trp into indole-3-acetaldoxime (IAOX) by an oxidoreductase, however, no orthologues have been identified in bacteria thus far, the oxidoreductase (K11812, K11813) was removed (8).

**Indole-3-Acetamide Pathway (IAM)**：

K00466, (K01426,K21801)

**Indole-3-Pyruvate Pathway (IPA)** beneficial bacteria:

(K03334,K14265,K00838,K16903), K04103, (K00128,K14085,K00149,K11817,K22417)

(K03334,K14265,K00838,K16903), K11816

**Tryptamine pathway (TAM):**

(K01593,K22433), (K00274,K11182), (K00128,K14085,K00149,K11817,K22417)

**Indole-3-acetonitrile pathway (IAN):**

K11868, K01501

K11868, (K01721,K20807), (K01426,K21801)

K11818, K11819, K11820, K11821, K01237, K01501

K11818, K11819, K11820, K11821, K01237, (K01426,K21801)

**REFERENCES**

1. Raux, E., Thermes, C., Heathcote, P., Rambach, A., & Warren, M. J. (1997). A role for Salmonella typhimurium cbiK in cobalamin (vitamin B12) and siroheme biosynthesis. *Journal of bacteriology*, *179*(10), 3202-3212..
2. Engelberts, J. P., Robbins, S. J., de Goeij, J. M., Aranda, M., Bell, S. C., & Webster, N. S. (2020). Characterization of a sponge microbiome using an integrative genome-centric approach. *The ISME journal*, *14*(5), 1100-1110.
3. Bulgarelli, D., Schlaeppi, K., Spaepen, S., Van Themaat, E. V. L., & Schulze-Lefert, P. (2013). Structure and functions of the bacterial microbiota of plants. *Annual review of plant biology*, *64*, 807-838.
4. Cook, S. D. (2019). An historical review of phenylacetic acid. *Plant and Cell Physiology*, *60*(2), 243-254.
5. Duca, D., Lorv, J., Patten, C. L., Rose, D., & Glick, B. R. (2014). Indole-3-acetic acid in plant–microbe interactions. *Antonie Van Leeuwenhoek*, *106*(1), 85-125.
6. Sun, S. L., Yang, W. L., Fang, W. W., Zhao, Y. X., Guo, L., & Dai, Y. J. (2018). The plant growth-promoting rhizobacterium Variovorax boronicumulans CGMCC 4969 regulates the level of indole-3-acetic acid synthesized from indole-3-acetonitrile. *Applied and environmental microbiology*, *84*(16), e00298-18.
7. Duca, D., Lorv, J., Patten, C. L., Rose, D., & Glick, B. R. (2014). Indole-3-acetic acid in plant–microbe interactions. *Antonie Van Leeuwenhoek*, *106*(1), 85-125.
8. Zhang, P., Jin, T., Kumar Sahu, S., Xu, J., Shi, Q., Liu, H., & Wang, Y. (2019). The distribution of tryptophan-dependent indole-3-acetic acid synthesis pathways in bacteria unraveled by large-scale genomic analysis. *Molecules*, *24*(7), 1411.
